# An Adaptive Exposure Fusion Method Using Fuzzy Logic and Multivariate Normal Conditional Random Fields

**DOI:** 10.3390/s19214743

**Published:** 2019-10-31

**Authors:** Yu-Hsiu Lin, Kai-Lung Hua, Hsin-Han Lu, Wei-Lun Sun, Yung-Yao Chen

**Affiliations:** 1 Department of Electrical Engineering, Ming Chi University of Technology, New Taipei 243, Taiwan; yhlin@mail.mcut.edu.tw; 2 Department of Computer Science and Information Engineering, National Taiwan University of Science and Technology, Taipei 106, Taiwan; hua@mail.ntust.edu.tw; 3 Graduate Institute of Automation Technology, National Taipei University of Technology, Taipei 106, Taiwan; t106618023@ntut.edu.tw (H.-H.L.); t104618028@ntut.edu.tw (W.-L.S.)

**Keywords:** fuzzy logic, intelligent vision sensing, exposure fusion, coarse-to-fine tuning, detail manipulation

## Abstract

High dynamic range (HDR) has wide applications involving intelligent vision sensing which includes enhanced electronic imaging, smart surveillance, self-driving cars, intelligent medical diagnosis, etc. Exposure fusion is an essential HDR technique which fuses different exposures of the same scene into an HDR-like image. However, determining the appropriate fusion weights is difficult because each differently exposed image only contains a subset of the scene’s details. When blending, the problem of local color inconsistency is more challenging; thus, it often requires manual tuning to avoid image artifacts. To address this problem, we present an adaptive coarse-to-fine searching approach to find the optimal fusion weights. In the coarse-tuning stage, fuzzy logic is used to efficiently decide the initial weights. In the fine-tuning stage, the multivariate normal conditional random field model is used to adjust the fuzzy-based initial weights which allows us to consider both intra- and inter-image information in the data. Moreover, a multiscale enhanced fusion scheme is proposed to blend input images when maintaining the details in each scale-level. The proposed fuzzy-based MNCRF (Multivariate Normal Conditional Random Fields) fusion method provided a smoother blending result and a more natural look. Meanwhile, the details in the highlighted and dark regions were preserved simultaneously. The experimental results demonstrated that our work outperformed the state-of-the-art methods not only in several objective quality measures but also in a user study analysis.

## 1. Introduction

All surroundings have a large dynamic range—the luminance of the highlight region might be over one hundred thousand times larger than that of the dark region. However, common cameras can capture a small portion of the dynamic range. If the exposure time is long, a detailed scene in a dark region can be captured. However, the content in the highlight region is lost because of over-saturation (over-exposure). By contrast, if the exposure time is short, the details in the dark region are lost because of under-exposure. Both are unacceptable. In addition, most traditional display devices only support 24 bit RGB (red, green, and blue) color images. In this case, representing all details of natural scenes on displays is a challenge. Displaying natural scenes as perceived through the human visual system becomes a difficult task; therefore, high dynamic range (HDR) techniques play a crucial role in vision-based intelligent systems. For example, smart sensors with HDR techniques enable high visual ability in environmental sensing, which can be used in intelligent traffic monitoring and vehicle-mounted sensors [1].

Different from another HDR technique called *tone mapping* [2,3], which requires larger bit depth of the image than a 24 bit depth per pixel, exposure fusion only requires several low dynamic range (LDR) images and can directly produce an LDR image which visually imitates the HDR scene with high quality. Exposure fusion methods bypass the procedure of generating the HDR images and directly fuse the best (i.e., visually meaningful with details) regions. However, capturing the appropriate regions from individual input images is not easy but critical in exposure fusion. Moreover, how to fuse different images seamlessly and to preserve the color consistency in local regions makes it more challenging. 

By fusing a bracketed exposure sequence, exposure fusion can effectively solve the problem of limited dynamic range because of single-shot imaging, i.e., capturing the scene with only a single exposure. Many exposure fusion methods have been proposed in the last decade. In the most relevant studies, the differently exposed images are assumed to be aligned perfectly when they are taken as input. Therefore, determining the appropriate pixel weights from individual images is the most essential step in exposure fusion. Mertens et al. [4] proposed evaluating three quality measures (i.e., image contrast, color saturation, and exposure brightness) as the reference to determine the contribution of a pixel to the final composite image. The Laplace pyramid is applied for multiscale blending to avoid artifacts such as seams and halos around edges. However, it is indeed important to preserve the details in highlight and dark regions simultaneously, but the method in Reference [4] does not fully address the issue. Compared with Reference [4], detail preservation is one of the main contributions of this work. 

Some recent exposure fusion methods are reviewed as follows. Jung and Ho [5] proposed an exposure fusion method which advocates the posterior probability-based blending of two exposed images for HDR image generation. To find the maximum a posteriori solution, the involved cost value takes the image gradient and chrominance components into account. Ancuti et al. [6] proposed a single-scale fusion approach, which simplifies the traditional pyramid-based multiscale fusion while preserving the majority of the information. Although in Reference [6], the fusion formula is similar to single-level naïve fusion, it demonstrates a close approximation result to the multiscale fusion. Kinoshita et al. [7] proposed an exposure fusion method based on automatic exposure compensation, where a luminance adjustment method was presented to improve the quality of the input multi-exposure images. Their work tries to solve the problem of how to determine the appropriate degree of exposure values so that the saturation regions of the fused image can be decreased. Liu and Zhang [8] proposed an underexposed image enhancement method using weighted multi-exposure fusion, where the optimal weights are determined by an energy function to preserve details and enhance edges. Hayat and Imran [9] presented a multi-exposure image fusion technique, which utilizes the dense scale-invariant feature transform (SIFT) descriptor to overcome the ghost artifact problem in exposure fusion. In Reference [10], Kinoshita and Kiya proposed a segmentation-based approach for luminance adjustment and enhancement, which can be applied in input differently exposed images to improve the quality of the final fused image. Ma et al. [11] presented a patch-decomposition-based exposure fusion method, where three components (signal strength, signal structure, and mean intensity) were analyzed from individual image patches. Ma et al. [12] proposed an exposure fusion method which can improve the image quality by optimizing the color multi-exposure image-fusion structural similarity index. 

Image noise arises another concern in exposure fusion. When the same scene is captured under different exposures, the corresponding levels of noise are also different. For example, noise is more likely to exist in the dark regions of the underexposed image. Moreover, using higher photosensibility (i.e., International Organization for Standardization (ISO) sensitivity) is likely to induce more noise. Such noise might be further amplified through the fusion process. To suppress excess noise and preserve the edge information, this work adopted the weighted guided image filter (WGIF) [13] on the weight maps. The WGIF is an edge-aware smoothing operator, which is widely used in many image processing areas, such as image de-hazing [14], image de-noising [15], image decomposition [16], and contrast enhancement [17]. Applying WGIF in the proposed enhanced fusion allows users to manipulate the degree of sharpness in a more appropriate way, and the details in the highlight/dark regions are better preserved.

After determining the optimal weights by the fuzzy-MNCRF model, this paper adopted the pyramid decomposition scheme for multi-scale fusion of differently exposed images. The concept of pyramid-based fusion is to first smooth and sub-sample all the input images repeatedly (according to how many levels we want) and then fuse them through individual levels of image pyramid (spatial scales). Applying the pyramid decomposition scheme creates a set of cascading versions of the input image, which is useful in extracting structures or features at multiple scales. In addition to image fusion, pyramid decomposition scheme is also applied in different topics, such as image filtering [18], dehazing [19], and image decolorization [20]. Compared to single-scale weighted averaging, multi-scale fusion provides more seamless and pleasant results. In view of the advantage of multi-scale decomposition, there are several representative multi-scale exposure fusion methods proposed recently. In Reference [21], the first stage is similar to an extension of Reference [4] by integrating with the weighted guided filter, and the second stage involves using the structure tensor to preserve the details in the bright/dark regions. In Reference [22], an edge-preserving smoothing pyramid, which is based on the gradient domain-guided image filter (GGIF) [23], is proposed to preserve the details in the brightest or darkest regions for multi-scale exposure fusion. In Reference [24], a multi-scale exposure fusion in YUV ((indicating luminance, chrominance, and chroma)) Color Space is proposed, which addresses the issue of the computational complexity of edge-preserving smoothing. Compared to the above methods, this work also presents a detail preservation scheme; moreover, we utilized the MNCRF model to fine-tune the weight maps (before the multi-scale fusion stage) for pleasing image quality. 

The rest of this paper is organized as follow. In Section 2, we briefly explain the motivation of combining fuzzy logic and the MNCRF model in fusion weighting. In Section 3, we present the proposed approach. In Section 4, we provide the experimental results and compare them with the existing state-of-the-art methods. Finally, we conclude the paper in Section 5.

## 2. Motivation of Integrating Fuzzy Logic with MNCRF Model

Because of its applicability and capability of handling non-numerical information, fuzzy logic has been applied to many image processing topics, such as fuzzy filtering [25], fuzzy segmentation [26], and fuzzy contrast enhancement [27]. Fuzzy logic also demonstrates its effectiveness in some recently proposed image fusion methods. Celebi et al. [28] applied fuzzy logic to determine fusion weights; only one input image was required, and the other exposed images are generated from the input image by histogram separation and histogram equalization techniques. Rahman et al. [29] proposed a multifocal image fusion method, where the fuzzy logic is used to determine the degree of focus for in-focus and out-of-focus data. 

**Figure 1:**
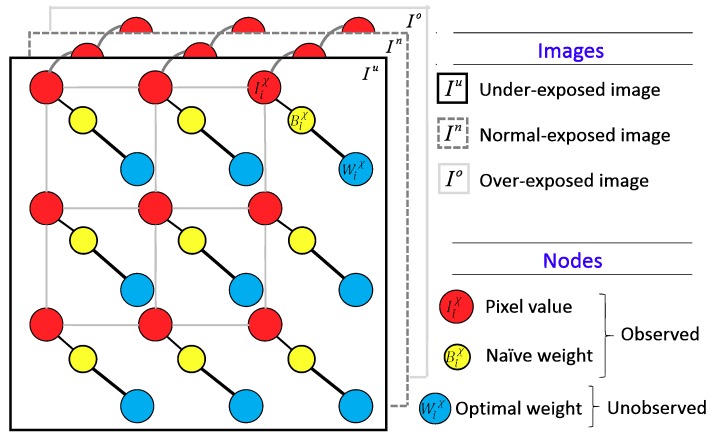
Undirected graph representation of the multivariate normal conditional random fields (MNCRF) model, where the blue nodes denotes the unknown desired random variables (RVs) in terms of the fine-tuned weights, the red nodes denotes the observable RVs in terms of the pixel values, and the yellow nodes denotes the observable RVs in terms of the naïve weights.

In References [28,29], the fusion weights were determined by unidirectional analysis using fuzzy logic. We explored some artifacts in the fused images that were output from their methods, especially in local color inconsistency. Chen et al. [30] presented an exposure fusion method, which uses a fuzzy-feedback loop to control the sharpness of fused images in a more appropriate way. The image quality was considerably improved using this method. However, the number of loops might increase computational complexity. To address this difficulty, we proposed a two-step sequence-based weighting procedure that uses fuzzy logic to determine the initial fusion weights and uses the multivariate normal conditional random fields (MNCRF) model [31] to fine tune the weights. The undirected graph of the MNCRF model is illustrated in Figure 1, where the linkages between nodes indicates the associated conditional dependency.

The MNCRF model is a scheme based on the stochastic process of multivariate vectors, which can encode contextual relationships among different random variables. It is widely applied in the areas which require excellent image quality or fine and precise details, such as image denoising [32], HDR map estimation [33], saliency detection [34], and object detection [35]. Therefore, this work utilized the MNCRF model to fine tune the weights. The proposed two-step weighting was based on our observation that a successful exposure fusion involves not only determining the weight according to individual pixel importance (i.e., weighting results of fuzzy inference system, FIS) but also considering the intra- and inter-image information simultaneously to maintain smoothness. 

## 3. Proposed Approach

**Figure 2:**
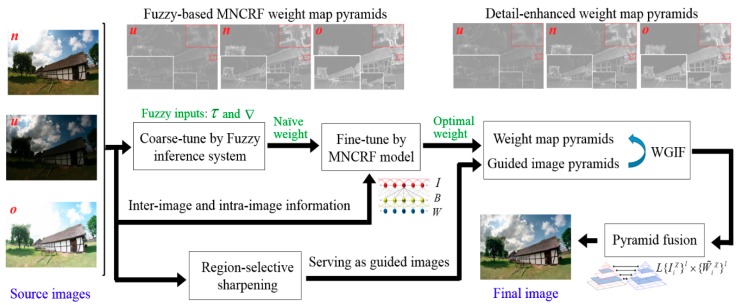
Overall framework of the proposed method, where *u*, *n*, and *o,* respectively, indicate under-exposure, normal-exposure, and over-exposure. For simplicity, we only show the pyramids of the red plane.

Throughout this paper, we use superscript χ={u,n,o} to denote different exposure levels: *u*, *n*, and *o,* respectively, indicate under-exposure, normal-exposure, and over-exposure. We use subscript i to denote the pixel position. Figure 2 describes the overall framework of the proposed approach. For simplicity but without loss of generality, we assumed that there were three input differently exposed images $I_{i}^{\chi }$.

### 3.1. Fuzzy-Based Pixel Weights Initialization

One of the most typical exposure fusion methods is the method proposed in Reference [4], which determines the pixel weights by considering different properties at the same time. However, we have found some artifacts, such as local hue inconsistency and slight seam effects, in Reference [4] which probably come from the imbalance among those properties.

The fuzzy inference system (FIS) provides a straightforward and efficient method for modeling complex systems through fuzzy variables. Because exposure fusion involves searching for portions with details from input images $I_{i}^{\chi }$ and blending them to construct an HDR-like scene, quality metrics are excellent indicators to determine the fusion weights. To measure quality, the color space was converted from RGB to YUV (indicating luminance, chrominance, and chroma) color space. The proposed FIS was based on our observation that regions containing well-exposed or large gradients play an essential role in the fusion stage. In this study, two quality metrics were entered as inputs in the FIS, well-exposedness (τ) and local pixel-visibility (*∇*), which are, respectively, defined as follows:

(1) $$\tau_{i}^{\chi }=\exp\left(\frac{-1}{2\sigma^{2}}{\left(Y_{i}^{\chi }-128\right)}^{2}\right)$$

and

(2) $${𝞩}_{i}^{\chi }=\max\left(\left|Y_{i}^{\chi }-Y_{j}^{\chi }\right|\right),j\in N_{4}\left(i\right)$$

where Y denotes the luminance value, and the symbol $N_{4}\left(.\right)$ denotes the 4 connected neighboring pixels. Normally, if the luminance value is closer to 128, the image has a more pleasant visual appearance and is worth a higher weight. Thus τ simulates this property by using a Gaussian curve. Moreover, *∇* simulates the directional derivative that is close to calculating the gradient value, where the maximum operation is exploited in comparing the intensity difference to decrease the computation cost. Table 1 constructs the fuzzy rule base for FIS, which is specified by observing massive images. After the defuzzification process, the initial pixel weight (B) can be expressed as follows:

(3) $$B_{i}^{\chi }=fuzzy_{i}^{\chi }\times {\left[\underset{\chi }{\sum }fuzzy_{i}^{\chi }\right]}^{-1}$$

where ${fuzzy}_{i}^{\chi }\;,\;\chi =\left\{u,n,o\right\}$ indicates the crisp output from the FIS.

### 3.2. Weight Fine-Tuned Using the MNCRF Model 

Fuzzy weighting allows us to efficiently extract both well-exposed regions and pixels containing strong local pixel-visibilities. Nevertheless, the color inconsistency problem, such as local hue inconsistency and seam effects, are not well-solved thus far, mainly because of two reasons: 1) the information in the UV channels is not considered yet; and 2) the inter- and intra-image relationships are not considered properly. Generating a high-quality HDR-like image is beyond weighting by the pixels’ importance separately. Apparently, the initial weights from the FIS are somehow unbalanced among current properties and lack of analyzing the mutual relationship among different input images simultaneously.

To address this problem, this study applied the MNCRF model to formulate the abovementioned information by treating $B_{i}^{x}$ as the naïve weight. Modeling the weight determination in MEF (multiple exposure fusion) is sensitive. To avoid over-adjustment, the relationship between the naïve weight and its corresponding desired pixel weight was assumed to be a zero-mean Gaussian distribution. Moreover, to take the spatial coherence into account, the relationship among the desired neighboring pixel weights in the local region was also assumed to be another zero-mean Gaussian distribution. In the MNCRF model, two matrices are defined:

(4) $$B=\left[\begin{array}{ccc} B_{1}^{o} & \cdots & B_{1}^{u}\\ \vdots & \ddots & \vdots \\ B_{N}^{o} & \cdots & B_{N}^{u} \end{array}\right]\;\mathrm{and}\;W=\left[\begin{array}{ccc} W_{1}^{o} & \cdots & W_{1}^{u}\\ \vdots & \ddots & \vdots \\ W_{N}^{o} & \cdots & W_{N}^{u} \end{array}\right]$$

where B is the naïve weight matrix, W is the corresponding MNCRF weight matrix, and N is the total number of pixels in an input image. This work adopts the maximum-a-posteriori (MAP) procedure to find the optimal W. 

#### 3.2.1. Inter-Image Relationships 

An N×N precision matrix Λ was designed to represent the inter-image relationships of B and W which can be expressed as Λ=U+V**.** The matrix U is a diagonal matrix. Thus, the inter-image exposure correlation (i.e., same pixel position, but from differently exposed images) was considered. If the exposedness values of a pixel position for the three differently exposed images are similar (i.e., $Y_{i}^{o}\approx Y_{i}^{n}\approx Y_{i}^{u}$), it implies that this position does not belong to an exposure-sensitive region and, thus, a more flexible modification of pixel weight can be presented in this pixel position. Therefore, the matrix U is defined as follows:

(5) $$U_{i,j}=\{\begin{array}{c} \exp\left[-\frac{\left|\tau_{i}^{o}-\tau_{i}^{n}\right|+\left|\tau_{i}^{n}-\tau_{i}^{u}\right|}{\sigma_{1}}\right]\;,\;if\;i=j\\ 0\;,\;\mathrm{otherwise} \end{array}$$

where (i,j) is the element position of a matrix, and $\sigma_{1}=1$ in this work. The matrix U is further normalized so that the largest entry value is equal to one.

The matrix V is a symmetric matrix, which considers the accumulated local hue continuity from the three input images. Because usually the spatially neighboring pixels have high probabilities of belonging to the same object, they have high chances of having similar exposedness, hue, and pixel weight. The MNCRF model should build a link between neighboring hue/luminance similarity and the output weights to alleviate the interference from noise and luminance variation. Therefore, the matrix V is defined as follows:

(6) $$V_{i,j}=\{\begin{array}{c} \underset{\chi }{\prod }\exp\left[-\frac{\left|\tau_{i}^{\chi }-\tau_{j}^{\chi }\right|\cdot \Delta UV_{ij}^{\chi }}{\sigma_{2}}\right]\;,\;if\;j\in N_{4}\left(i\right)\\ 0\;,\;\mathrm{otherwise} \end{array}$$

where $\sigma_{2}$ is set as 1, and $\Delta UV_{ij}^{\chi }$ is the chrominance difference defined in the UV color plane:

(7) $$\Delta UV_{i,j}^{\chi }=\sqrt{{\left(U_{i}^{\chi }-U_{j}^{\chi }\right)}^{2}+{\left(V_{i}^{\chi }-V_{j}^{\chi }\right)}^{2}}/255$$

#### 3.2.2. Intra-Image Relationships

An N×N precision matrix ∑ was designed to represent the intra-image relationships of B and  W which can be expressed as ∑=P+Q. The matrix Q is a symmetric matrix which takes neighboring color similarity into account. The color similarity (CS) index of the adjacent pixel pair (i,j) is defined as follows:

(8) $${\mathrm{CS}}_{i,j}^{\chi }=\{\begin{array}{c} \frac{1}{\pi }\left[\frac{\gamma }{{\left(\Delta YUV_{i,j}^{x}\right)}^{2}+\gamma^{2}}\right]\\ 0\;,\;\mathrm{otherwise} \end{array}\;,\;\mathrm{if}\;j\in N_{4}\left(i\right)$$

where γ is set within the range of [0.4,0.6]. Similar to Equation (7), $\Delta YUV_{ij}^{\chi }$ is the color difference defined in the YUV color space:

(9) $$\Delta YUV_{ij}^{\chi }=\sqrt{{\left(Y_{i}^{\chi }-Y_{j}^{\chi }\right)}^{2}+{\left(U_{i}^{\chi }-U_{j}^{\chi }\right)}^{2}+{\left(V_{i}^{\chi }-V_{j}^{\chi }\right)}^{2}}/255$$

The CS index is constructed based on a Cauchy function, which is also a bell-shape function (as is the Gaussian function). However, as the color difference increases, the Cauchy function decreases more dramatically than the Gaussian function, which matches our observation on the weight adjustment. If the neighboring pixels have high color coherence, their linkage in the MNCRF model should be strong. Therefore, the matrix Q is defined as follows:

(10) $$Q_{i,j}=\{\begin{array}{c} \underset{\chi }{\prod }{\mathrm{CS}}_{i,j}^{\chi }\;,\;\mathrm{if}\;j\in N_{4}\left(i\right)\\ 0\;,\;\mathrm{otherwise} \end{array}$$

The matrix P is a diagonal matrix, which takes the intra-image correlation into account to maintain the regional smoothness in the final fused image. If a pixel position has high color similarity to its four-neighboring pixels at all the three input images, then these pixels have a high possibility of belonging to the same object. Accordingly, the accumulation of both the CS and well-exposedness values is considered. Therefore, the matrix P is defined as follows:

(11) $$P_{i,j}=\{\begin{array}{c} \underset{j^{\prime }\in N_{4}\left(i\right)}{\sum }\left(\underset{\chi }{\prod }{\mathrm{CS}}_{i,j^{\prime }}^{\chi }\right)+\underset{\chi }{\sum }\tau_{i}^{\chi }\;,\;\mathrm{if}\;i=j\\ 0\;,\;\mathrm{otherwise} \end{array}$$

Derived from Reference [36], searching for the optimal fusion weights could be viewed as solving a MAP problem as follows:

(12) $$\hat{W}=\arg\underset{w}{\max}\exp\left(Tr\left(-W^{T}\wedge B-\frac{1}{2}W^{T}\sum W\right)\right)$$

where Tr(.) denotes the trace operator. The optimal W of the MNCRF model can be expressed as follows:

(13) $$\hat{W}=-{\left(P+Q\right)}^{-1}\left(U+V\right)B$$

where each column of W indicates the 1D representation of a weight map. 

**Figure 3:**
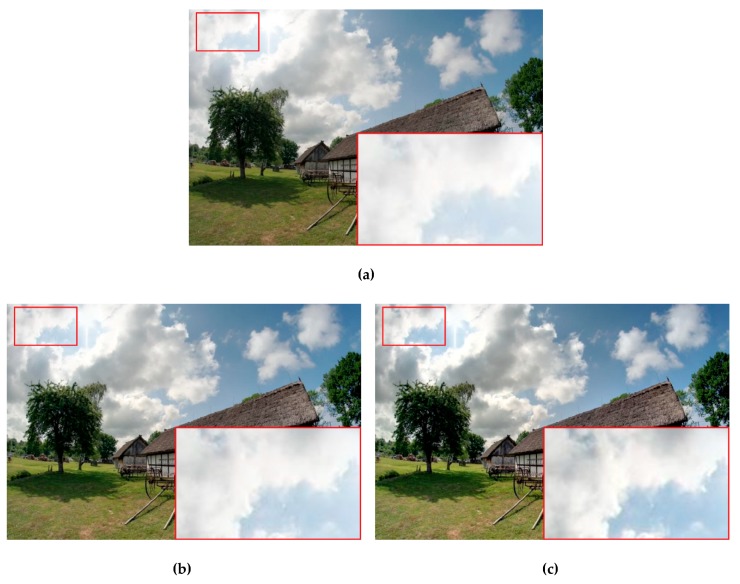
Visual comparison of the exposure fusion results from different weighting processes. (**a**) Well-exposedness only; (**b**) both the well-exposedness and local pixel-visibility using fuzzy logic; (**c**) the inter- and intra-image information using the proposed fuzzy-MNCRF model. For this example, the three input differently exposed images are shown in Figure 4.

As depicted in the enlarged region of the cloud in Figure 3, the local hues from the differently exposed images and the dark-to-bright gradients were transferred more smoothly in the fused image using the proposed method (comparison among Figure 3a–c). Meanwhile, the details were preserved more completely because of the combination of FIS with the MNCRF model.

### 3.3. Enhanced Multiscale Fusion with Region-Selective WGIF-Based Sharpening

Because each differently exposed image only contains a portion of dynamic range, there are three common major challenges in the fusion stage: edge-preserving, halo effects, and gradient reversal. To address these problems, we propose an enhanced multiscale fusion that utilizes the weighted guided image filter (WGIF) technique as follows. 

For the edge-preserving problem and only considering that the image gradients cannot completely represent the structural edges because these problems are scale-variant: a large gradient might not be an essential edge of the entire image, whereas a small gradient might be essential to a local region. In Reference [4], it was proven that pyramid representation is excellent at handling the edge-preserving decomposition problem with multiscale difference. Unlike the study in Reference [4], we used WGIF in two separate places to enhance the fine details. First, with regards to the structure-transferring property of WGIF, we added a preprocessing step in generating the guided images. Normally, the guided image is the input image itself. However, because WGIF can transfer the structural edges from the guided image to the input image, a region-selective sharpening (RSS) scheme was used to enhance the details of the guided image:

(14) $$BP_{i}^{\chi }=\mathrm{WGIF}\left(\mathrm{input}:I_{i}^{\chi },\mathrm{guided}:I_{i}^{\chi }\right)$$

and

(15) $$DP_{i}^{\chi }=n\times \eta_{i}\times \left(I_{i}^{\chi }-BP_{i}^{\chi }\right)+I_{i}^{\chi }$$

where base plane $BP_{i}^{\chi }$ is the WGIF result which has mostly homogeneous regions with edges inherited from $I_{i}^{\chi }$. Detail-enhanced plane $DP^{\chi }$ denotes the RSS result which has the same homogeneous regions as $I_{i}^{\chi }$ but more enhanced details in texture regions. In some works, $BP_{i}^{\chi }$ and $\left(I_{i}^{\chi }-BP_{i}^{\chi }\right)$ are referred to as the base layer (containing large-scale variations) and the detail layer (containing small-scale details), respectively. Parameter n is the boosting coefficient (n is suggested to range from five to ten), and $\eta_{i}$. was adopted from Reference [13] which is an edge-aware function used to distinguish the flat region from the texture region. 

The WGIF is a local linear filter. Compared to other edge-preserving filter, such as bilateral filter, WGIF has better protection against the artifacts of halo and gradient reversal. Here, ${\left\{{\hat{W}}_{i}^{\chi }\right\}}^{l}$ and ${\left\{DP_{i}^{\chi }\right\}}^{l}$, respectively, denote the Gaussian pyramids of the fuzzy-MNCRF weight map and the sharpened image, where *l* is the number of pyramid levels. According to the property of WGIF, the primary details of $DP_{i}^{\chi }$ are transferred to ${\hat{W}}_{i}^{\chi }$ at different pyramid levels through

(16) $${\left\{{\tilde{W}}_{i}^{\chi }\right\}}^{ı}=\mathrm{WGIF}\left(\mathrm{input}:{\left\{{\hat{W}}_{i}^{\chi }\right\}}^{ı}\;,\;\mathrm{guided}:{\left\{DP_{i}^{\chi }\right\}}^{ı}\right)$$

Then, the detail-enhanced weight pyramid ${\left\{{\tilde{W}}_{i}^{\chi }\right\}}^{l}$ is fused with the Laplacian pyramid of the differently exposed images ($L{\left\{I_{i}^{\chi }\right\}}^{l}$) at individual pyramid levels:

(17) $$L{\left\{{\tilde{I}}_{i}^{\chi }\right\}}^{ı}=\sum_{\chi }L{\left\{I_{i}^{\chi }\right\}}^{ı}\times {\left\{{\tilde{W}}_{i}^{\chi }\right\}}^{ı}$$

The final synthesized image is reconstructed by collapsing the pyramid of $L{\left\{I_{i}^{\chi }\right\}}^{l}$. 

## 4. Experimental Results and Discussions

**Figure 4:**
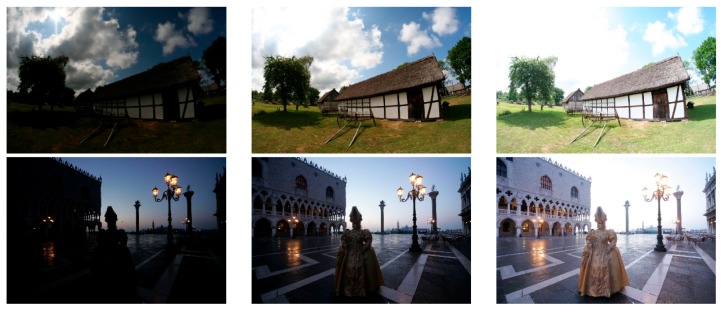

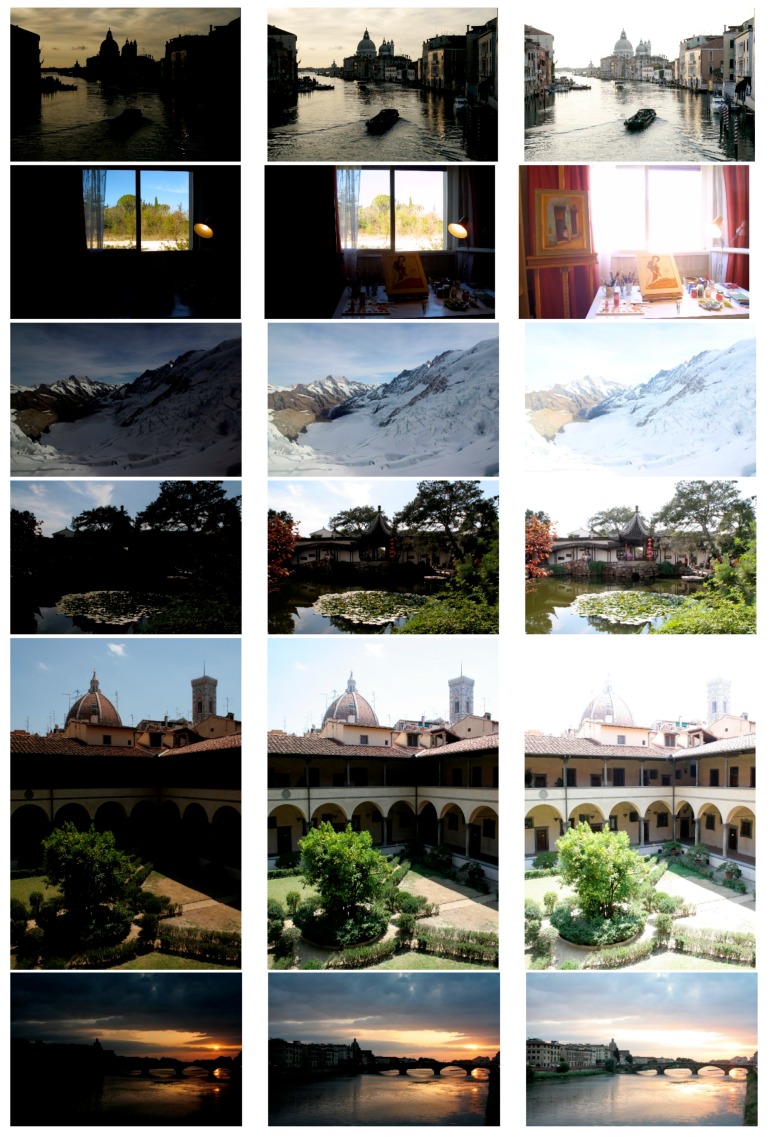
Eight test image sequences. (From top to bottom) Cottage, Masked Lady, Grand Canal, Studio, Mountains, Chinese Garden, Laurentian Library, and Arno River. The left-column, middle-column, and right-column images show the under-exposure, normal-exposure, and over-exposure images, respectively.

To evaluate the performance of the proposed method, it was compared with the four recent methods in Reference [5] (2013), Reference [6] (2017), Reference [28] (2015), and Reference [23] (2015). Eight test image sequences were selected from public databases [37,38], and each of them contained three exposure levels, as shown in Figure 4. Quality measures are the objective tools which help us to quantitatively evaluate the performance among different methods. In this paper, we selected five image quality measures described as follows.

### 4.1. Comparison of the Objective Quality Measures

The first quality measure is the Contrast and Sharpness Measurement Index (CSMI) introduced in [39]. The human visual system (HVS) captures wider dynamic range than a camera, which allows people to perceive details in every part of a real-world scene. Whereas in exposure fusion methods, normally the details in highlight and shadow regions are difficult to be preserved because of the limited dynamic range using a single shot. In CSMI, the contrast degree is evaluated by considering the difference between foreground and background using the logarithmic image processing operator, and the sharpness degree is evaluated by considering the boundaries between different zones using the wavelet decomposition. Therefore, the CSMI value is closely correlated to the HVS property which reflects people’s perceptions. Table 2 lists the resulting CSMI values of the four methods. As shown in the bottom row of Table 2, average CSMI values achieved by the five methods are respectively 5.3916 (method in Reference [5]), 8.3436 (method in Reference [6]), 8.2355 (method in Reference [28]), 8.5081 (method in Reference [23]), and 8.6860 (proposed method). Although the proposed method did not obtain the highest CSMI value in every test image sequence (e.g., the test images Mountains and Arno River), the comparison of the average CSMI values validated that the proposed method can effectively maintain the details’ sharpness and great contrast. 

The second quality measure is the image entropy value, which can be expressed as:

(18) $$\frac{1}{3}\underset{\rho =R,G,B}{\sum }\left(-\overset{255}{\underset{i=0}{\sum }}P\left(x_{i}^{\rho }\right)\log P\left(x_{i}^{\rho }\right)\right)$$

where i is the intensity levels of each color channel, $P\left(x_{i}^{\rho }\right)$ is the probability of a pixel with the intensity i, and ρ indicates one of the RGB channels. Entropy is a no-reference image quality assessment scheme, and the degree of Entropy indicates the richness of information content shown in a fused image. Therefore, in some works such as [28] and [29], Entropy is adopted to represent the level of detail-preserving ability. Normally for the highlight region of an over-exposed image and the shadow region of an under-exposed image, the detailed information is almost lost, which leads to a low Entropy value. However, a successful exposure fusion method should be able to extract the fine details form several differently exposed images and to present sufficient and high-quality details in all regions of the output image. Table 3 lists the resulting entropy values of the four methods. As shown in the bottom row of Table 3, the average entropy values achieved by the four methods were, respectively, 7.4047 [5], 7.5391 [6], 7.4229 [28], 7.4140 [23], and 7.6088 (our proposed method). Although the proposed method does not obtain the highest entropy value in every test image sequence (e.g., the test images Masked Lady, Grand Canal, Mountains, Arno River, and Studio), the comparison of the average image entropy values demonstrated that our approach can preserve the details of a natural scene to the greatest extent.

The third quality measure is specifically designed for the exposure fusion methods which is called the multi-exposure fusion structural similarity (MEF-SSIM) index [40]. Different from the original SSIM index that requires only a single reference image, the MEF-SSIM index aims to evaluate the ability of preserving information from the multiple input images at each pixel position. Moreover, the contrast and structure components of local image patches were also analyzed and taken into account when formulating the MEF-SSIM index. Table 4 presents the results of the MEF-SSIM values of the four methods. Promisingly, the proposed method demonstrates the superior ability to maintain the perception-based structural similarity from the results shown in Table 4. Among the eight test images, the MEF-SSIM scores of our approach were all higher than 0.9 except for the image *Studio* (but in this image, our score was still the highest of the four methods). In addition, the proposed method outperformed other comparative methods in every test image sequence. The average MEF-SSIM values achieved by the four methods were respectively 0.8344 [5], 0.8914 [6], 0.8500 [28], 0.829 [23], and 0.9415 (proposed method). 

In addition, Table 5 and Table 6 show the comparison results of two other objective metrics: a feature-enriched blind image quality evaluator called IL-NIQE [41] and a no-reference quality metric called NIQMC [42]. For the IL-NIQE metric, it is an opinion-unaware blind image quality assessment which is based on integrating several image statistics such as texture, color, and contrast. The IL-NIQE value reflects the naturalness of the fused image, and a lower IL-NIQE value indicates a more natural look. For the NIQMC metric, it is a no-reference and blind image quality assessment of contrast distortion, which is based on calculating the entropy of particular regions with maximum information. The NIQMC value reflects the contrast distortion of the fused image, and a higher NIQMC value indicates a more pleasing visual quality with better clarity. The average IL-NIQE values achieved by the four methods were 19.3959 [5], 18.7395 [6], 18.4621 [28], 19.6196 [23], and 17.8119 (proposed method). The average NIQMC values achieved by the four methods were, respectively, 4.9102 [5], 5.2867 [6], 5.0640 [28], 5.3400 [23], and 5.4606 (proposed method). As shown in Table 5 and Table 6, due to the combination of MNCRF, fuzzy, and WGIF-based enhancement, this work achieved the best average scores in both IL-NIQE and NIQMC metrics.

Furthermore, for the comparison of computational performance, the average processing times required to produce an image with a size of 870 × 578 were 7.1421 s [5], 1.9803 s [6], 5.8957 s [28], 1.0311 s [23], and 6.3402 s (proposed method). All methods were written in MATLAB and were implemented in the Windows 7 operating system with 3.2 GHz CPU. For the method in Reference [23], because it was a single-image enhancement method (we applied Reference [23]’s method in the normal-exposed image), it required the least processing time. For the proposed method, although combining the MNCRF model and the fuzzy-based weights initialization increased the computation cost, this work demonstrated superior image quality in the output fused images. 

### 4.2. Visual Comparison and User Study Analysis

**Figure 5:**
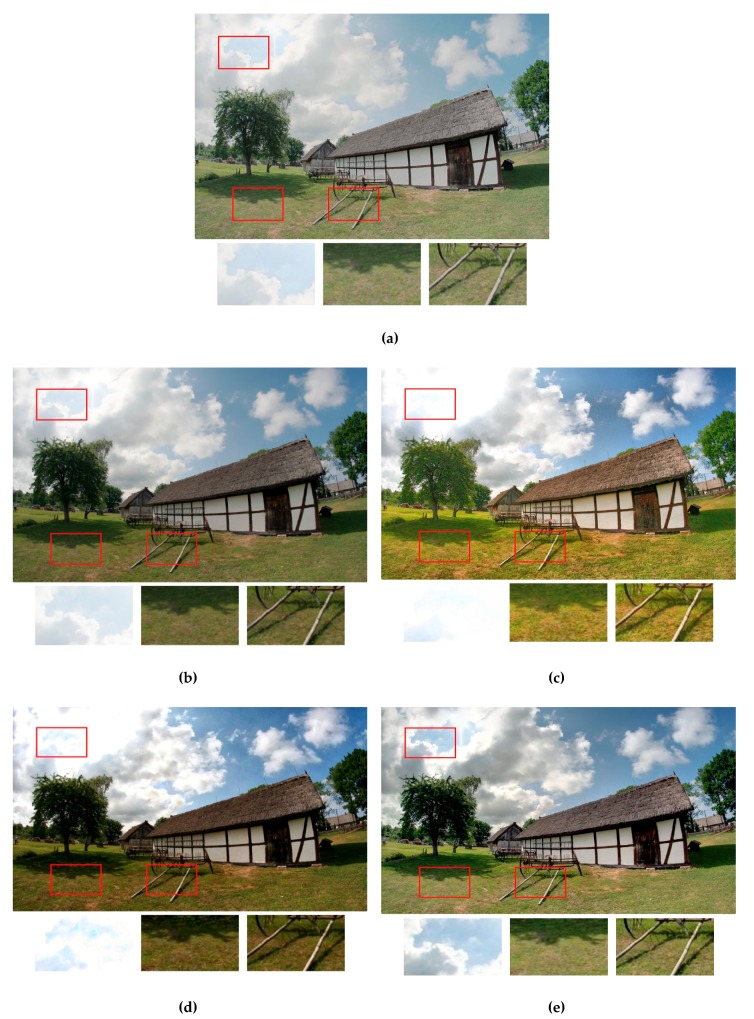
Visual comparison of the exposure fusion results using the test image Cottage. (**a**) Results from using the method in Reference [5]. (**b**) Results from using the method in Reference [6]. (**c**) Results from using the method in Reference [28]. (**d**) Results from using the method in Reference [23]. (**e**) Results from the proposed method. The enlarged versions of the red rectangles are provided to illustrate the subtle differences.

**Figure 6:**
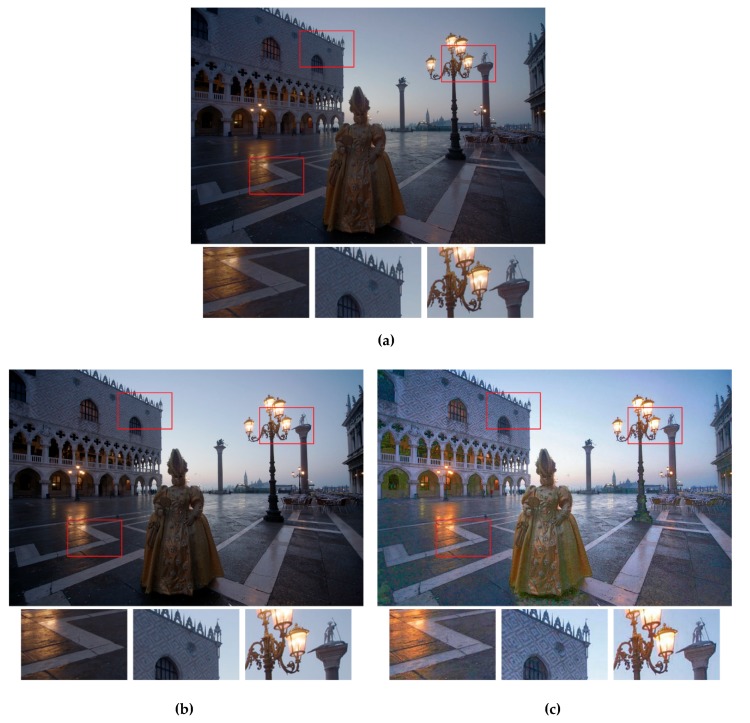

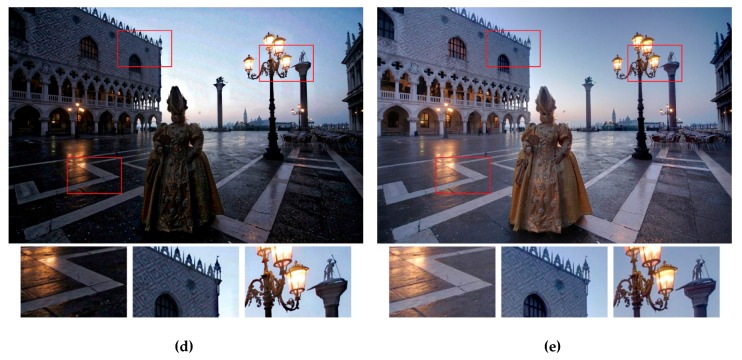
Visual comparison of the exposure fusion results using the test image *Masked Lady*. (**a**) Results from the method in Reference [5]. (**b**) Results from the method in Reference [6]. (**c**) Results from the method in Reference [28]. (**d**) Results from the method in Reference [23]. (**e**) Results from the proposed method. The enlarged versions of the red rectangles are provided to illustrate the subtle differences.

**Figure 7:**
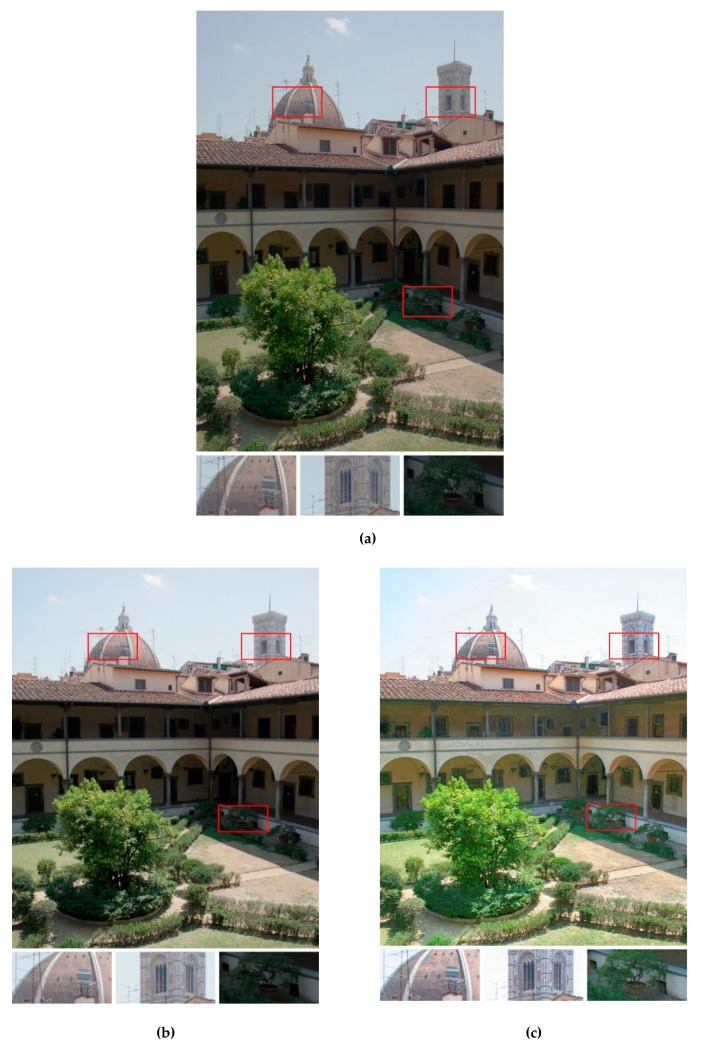

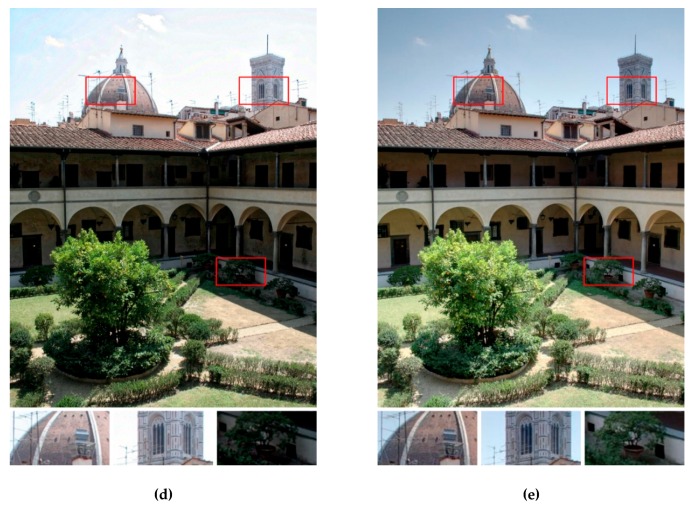
Visual comparison of the exposure fusion results using the test image *Laurentian Library*. (**a**) Results from the method in Reference [5]. (**b**) Results from the method in Reference [6]. (**c**) Results from the method in Reference [28]. (**d**) Results from the method in Reference [23]. (**e**) Results from the proposed method. The enlarged versions of the red rectangles are provided to illustrate the subtle differences.

In addition, to employ the objective quality measures, Figure 5, Figure 6 and Figure 7 provide the qualitative visual comparisons among the five methods. Putting the output fused images from different methods side by side allowed us to see the subtle but essential differences between our proposed strategy and the other exposure fusion methods.

Figure 5 shows the exposure fusion results using the test image Cottage. For the results for the Reference [5] (Figure 5a) method, the overall chrominance was somehow faded and lacked contrast. Moreover, the detailed textures, e.g., the details in the grass area were lost. This is consistent with the results shown in Table 2, where the CSMI value of this fused image (7.1133 in Figure 5a) was much lower than those of the other four images (9.3121 in Figure 5b, 9.4548 in Figure 5c, 9.2748 in Figure 5d, and 9.4681 in Figure 5e). For the result of the Reference [6] (Figure 5b) method, although the dynamic contrast was stretched, the color vividness was lost during the fusion process. For the results of the Reference [28] (Figure 5c) method, the top-left corner of the fused image was apparently over-exposed without preserving the details. This was because, when calculating the pixel weights of each input image, the weights were determined only through analyzing each single image without considering the inter-image relationships among each other. In this example, comparison among the sky regions from the different methods underlines our strategy of integrating the MNCRF model with fuzzy logic. In the sky region of the proposed method (Figure 5e), high-luminance, middle-luminance, and low-luminance pixels all appeared with very smooth gradients, and the WGIF-based enhanced fusion preserved the details. Therefore, a visually pleasing HDR-like image was generated.

Figure 6 shows the exposure fusion results using the test image Masked Lady. For the result of the Reference [5] (Figure 6a) method, the overall brightness was not enough. For example, the reflected light on the stone floor (the left enlarged image patch) was not as clear as the results shown in Figure 7c,d, and the texture of the wall (the center enlarged image patch) was vague. Similar phenomena occurred in the results of the Reference [6] method (Figure 6b). In both Figure 6a,b, the dynamic ranges of the fused images were not well stretched and were dim so that the details in the shadow regions of the scene were hardly preserved. For the result of the Reference [28] method (Figure 6c), the entire dynamic range was broadened through fusing the input images. For example, each window along the first-floor corridor can be seen. However, the overall chrominance was somehow greenish as shown in the clothes of the lady and the first-floor corridor. Moreover, the color of the lamp post (the right enlarged image patch) was unnatural. This reflects the difficulty of determining the appropriate pixel weights which can generate accurate colors and natural-looking images at the same time. For the result of the method in Reference [23] (Figure 6d), some white noise-like dots can be seen on the floor. The result of the proposed method (Figure 6e) outperformed the other methods in that not only were the relative contrast well preserved, but the global chrominance was pleasing and presented a more natural illumination of the real scene. Not accidentally, from the MEF-SSIM results shown in Table 4 (0.7878 in Figure 5a, 0.8628 in Figure 5b, 0.8467 in Figure 5c, 0.9245 in Figure 5d, and 0.9345 in Figure 5e), our method apparently overwhelming outperformed the others.

Figure 7 shows the exposure fusion results using the test image Laurentian Library. For the result of the method in Reference [5] (Figure 7a), the weighting process did not extract sufficient information from the normal-exposed image and the over-exposed image. Therefore, the highlight region such as the sky was not bright enough, and the details of the shadow region such as grass (the right enlarged image patch) were sacrificed. For the result of the method in Reference [6] (Figure 7b), the overall luminance was brighter than the result of the method in Reference [3]; however, the contrast was not stretched and the details of the grass region were still unclear. For the result of the method in Reference [28] (Figure 7c), the pixels of the input over-exposed image seemed to dominate the final fused image. Therefore, the details of the sky region were lost, and the color gamut was not wide. Moreover, the boundary between the sky and the tower (the center enlarged image patch) was unnatural and not smooth. For the result of the method in Reference [23] (Figure 7d), although the details are enhanced, the output image still lacked detail information from other differently exposed images. Moreover, while the details were enhanced, the noise was also amplified which led to some artifacts of unnatural color gradients shown in the sky. For the result of our work (Figure 7e), because the enhanced multiscale fusion with region-selective sharpening was utilized, the details of both highlights (e.g., sky and tower) and shadow (e.g., grass) were well preserved. Simply determining pixel weights by analyzing each image separately (by the fuzzy logics) was not enough to generate a high-quality HDR image. Combining the MNCRF model and fuzzy logic can modify the weights significantly. Furthermore, applying WGIF in the multiscale fusion enhanced the details in the bright/dark regions while avoiding over-amplifying the noise. From the comparison results shown in Table 2, Table 3 and Table 4, in this test image, the proposed method completely outperformed the other four methods in terms of CSMI, entropy, and MEF-SSIM.

**Figure 8:**
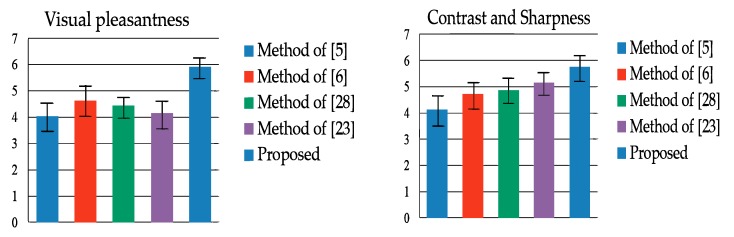
Results of the subjective test in terms of visual pleasantness and contrast/sharpness (average/standard deviation of the scores).

For the subjective evaluation, we invited 30 (15 male and 15 female) participants to conduct a visual quality test. The participants were asked to rate the visual pleasantness and the contrast/sharpness of each image. The visual pleasantness score indicates the participants’ preference. The contrast/sharpness score indicates whether the output fused image preserved clear details and edge information but was not unnaturally sharp. The scores ranged from 1 to 7, where score 1 indicated “unsatisfactory” and score 7 indicated “excellent.” Applying the MNCRF model to fine tune the weight maps enabled local color consistency and a wider range of color detail with more contrast because both intra- and inter-image information can be considered. Applying WGIF in the multiscale fusion ensures detail preservation while avoiding unpleasant noise. From the subjective user study results (summarized in Figure 8), the proposed method significantly outperformed the other four methods, especially in the aspect of visual pleasantness.

**Figure 9:**
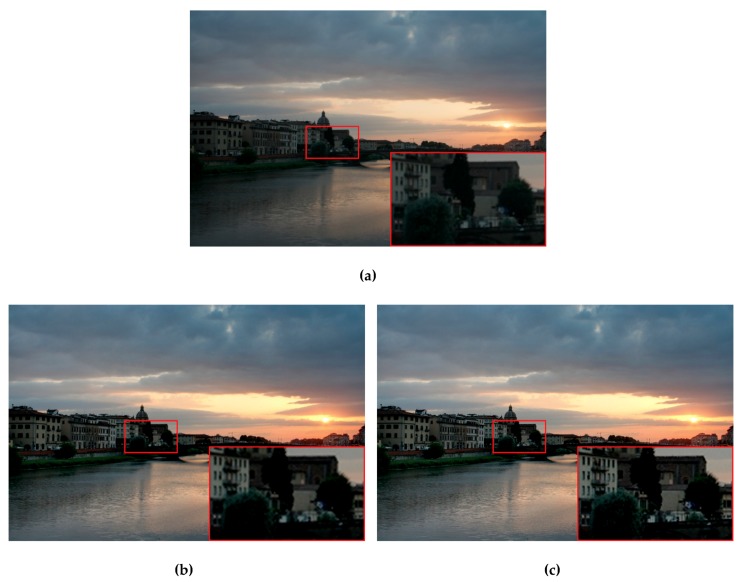
Illustration of the effectiveness of the proposed enhanced multiscale fusion using the test image Arno River. (**a**) Fusion result where the boosting coefficient equaled one. (**b**) Fusion result where the boosting coefficient equaled five. (**c**) Fusion result where the boosting coefficient equaled ten.

**Figure 10:**
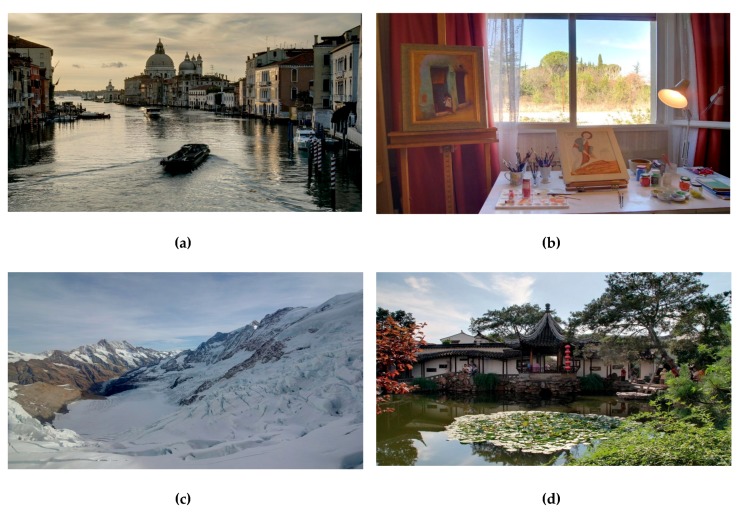
Results of the proposed method using the remaining test images. (**a**) Result of the test image Grand Canal. (**b**) Result of the test image Studio. (**c**) Result of the test image Mountains. (**d**) Result of the test image Chinese Garden.

**Figure 11:**
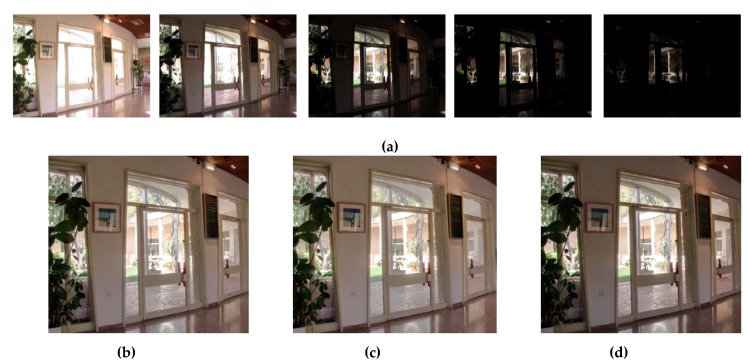
Results of the proposed method using different numbers of differently exposed images. (**a**) Sequence of differently exposed images (from Reference [23]). (**b**) Results of using the first three differently exposed images in (**a**). (**c**) Results of using the first four differently exposed images in (**a**). (**d**) Result of using all the differently exposed images in (**a**).

To demonstrate the effectiveness of the proposed enhanced multiscale fusion, Figure 9 illustrates an example for visual comparison. There are two merits of the proposed enhanced multiscale fusion. First, in exposure fusion, the extracted details are required to be enhanced to increase detail clarity. Second, in many computational photography applications, it is usually desirable to freely manipulate the sharpness level of the details in the fused image. As depicted in the enlarged region of building in Figure 9b,c, the proposed enhanced multiscale fusion effectively improves the sharpness and preserves the structural edges. By integrating WGIF in the weight pyramid and using a controllable boosting coefficient shown in Equation (15), detail manipulation is achieved without visual artifacts. Figure 10 shows the results of the proposed method using the remaining test images. To enrich the experimental results, we also tested the performance by fusing more than three images using the proposed method, as shown in Figure 11. For the case of fusing four images (Figure 11c), there were four initial weight maps generated by the fuzzy weighting process. Then, both the naïve weight matrix (the matrix B) and the MNCRF weight matrix (the matrix W) became N×4 matrices, and the maximum-a-posteriori procedure in Reference [36] was still able to find the optimal W. For the case of fusing five images (Figure 11d), it is similar to the case of fusing four images.

## 5. Conclusions

In this paper, we present a novel exposure fusion method which integrates fuzzy logic and the MNCRF model to achieve an adaptive coarse-to-fine weight determination process. Determining optimal pixel weights from individual bracketed images is a primary challenge for exposure fusion. Obviously, the highlights in an over-exposed image tend to be blown out and almost white; conversely, the shadows in an under-exposed image tend to be flat and almost black. In both cases, the information on the detail and color is lost. However, simply determining pixel weights by analyzing each image separately is not enough to generate a high-quality HDR image. To address this difficulty, in addition to the coarse initial weighting conducted by applying fuzzy logic, this work incorporated the MNCRF model into the fine-tuning stage to take inter-image information into account. Moreover, a multiscale enhanced fusion scheme was proposed to blend images with edge-preserving and even edge-enhancing. Exposure fusion methods are essential to applications involving human–computer interaction and intelligent vision sensing, because, actually, the human visual system has a much wider dynamic range than a common optical sensor. The experimental results validated the superiority of the proposed method in terms of objective quality measures (CSMI, entropy, MEF-SSIM, IL-NIQE, and NIQMC) and subjective user evaluation, compared with the state-of-the-art methods. For future work, we plan to investigate the possibility of fusing large-exposure-ratio images using the proposed method (especially, if there are only two to-be-fused images.) Fusing large-exposure-ratio images is an interesting problem mentioned in Reference [43], because, in this case, the highlight regions in the under-exposed image might be darker than the shadow regions in the over-exposed images.

## Figures and Tables

**Table 1:** Fuzzy rule base of fusion weight initialization.

	Exposedness	Low	Medium	High
Pixel-visibility				
**Low**		L	M–L	M
**Medium**		M–L	M	M–H
**High**		M	M–H	H

L: low, M–L: medium-low, M: medium, M–H: medium-high, H: high

**Table 2:** Comparison of the five methods in terms of image CSMI [39].

	Method	Method of [5]	Method of [6]	Method of [28]	Method of [23]	Proposed
Image						
Cottage		7.1133	9.3121	9.4548	9.2748	9.4681
Masked Lady		3.7015	7.2641	7.0846	7.3124	7.3579
Grand Canal		7.1152	10.3240	10.7570	10.8148	10.8990
Studio		2.9509	5.2579	4.9159	5.1922	5.2857
Mountains		3.9031	4.6651	4.2465	4.5157	4.6059
Chinese Garden		9.1185	14.9413	14.2991	15.2331	16.3545
Laurentian Library		6.6231	10.9506	10.3437	10.9220	11.0333
Arno River		2.6075	4.0337	4.7827	4.7998	4.4832
**Average**		**5.3916**	**8.3436**	**8.2355**	**8.5081**	**8.6860**

Red numbers indicates the best entropy value of each row.

**Table 3:** Comparison of the five methods in terms of image entropy.

	Method	Method of Reference [5]	Method of Reference [6]	Method of Reference [28]	Method of Reference [23]	Proposed Method
Image						
Cottage		7.7012	7.7878	7.7661	7.6545	7.9077
Masked Lady		7.2523	7.4959	7.6040	7.5180	7.5058
Grand Canal		7.4973	7.5888	7.8199	7.7353	7.7493
Studio		7.5419	7.5544	7.4239	6.6548	7.5542
Mountains		6.5399	6.7724	6.5173	7.3431	6.9725
Chinese Garden		7.6596	7.8087	7.5363	7.3059	7.8311
Laurentian Library		7.6578	7.8620	7.5551	7.5777	7.8934
Arno River		7.3875	7.4426	7.1607	7.5230	7.4567
**Average**		**7.4047**	**7.5391**	**7.4229**	**7.4140**	**7.6088**

Red numbers indicate the best entropy value for each row.

**Table 4:** Comparison of the five methods in terms of MEF-SSIM (multi-exposure fusion structural similarity) [40].

	Method	Method of Reference [5]	Method of Reference [6]	Method of Reference [28]	Method of Reference [23]	Proposed Method
Image						
Cottage		0.8617	0.8875	0.8672	0.8967	0.9456
Masked Lady		0.7878	0.8628	0.8467	0.9245	0.9345
Grand Canal		0.8483	0.8695	0.8314	0.8247	0.9424
Studio		0.7095	0.7659	0.6926	0.8762	0.8454
Mountains		0.9187	0.9721	0.9292	0.8621	0.9824
Chinese Garden		0.8146	0.9521	0.8693	0.9081	0.9640
Laurentian Library		0.8523	0.9104	0.8820	0.9354	0.9625
Arno River		0.8823	0.9110	0.8813	0.8358	0.9548
**Average**		**0.8344**	**0.8914**	**0.8500**	**0.8829**	**0.9415**

Red numbers indicate the best MEF-SSIM value for each row.

**Table 5:** Comparison of the five methods in terms of IL-NIQE [41].

	Method	Method of Reference [5]	Method of Reference [6]	Method of Reference [28]	Method of Reference [23]	Proposed Method
Image						
Cottage		15.7939	15.8029	16.9300	16.0214	17.2690
Masked Lady		20.1251	19.4580	18.4810	18.9070	17.8545
Grand Canal		17.7827	19.0877	17.8064	19.3340	16.2722
Studio		24.6146	23.2614	21.3269	26.2480	20.3581
Mountains		19.6483	19.2559	19.0458	17.0945	18.1613
Chinese Garden		13.7786	13.9000	14.4244	15.4630	14.2424
Laurentian Library		17.8698	17.2073	16.7130	19.0272	17.3954
Arno River		25.5544	21.9432	22.9693	24.8623	20.9425
**Average**		**19.3959**	**18.7395**	**18.4621**	**19.6196**	**17.8119**

Red numbers indicate the best IL-NIQE value for each row.

**Table 6:** Comparison of the five methods in terms of NIQMC [42].

	Method.	Method of Reference [5]	Method of Reference [6]	Method of Reference [28]	Method of Reference [23]	Proposed Method
Image						
Cottage		5.3764	5.7228	5.4693	5.7325	5.7211
Masked Lady		4.6466	5.0959	5.0698	5.2302	5.3977
Grand Canal		5.374	5.4604	5.4632	5.7162	5.3884
Studio		5.0849	5.4033	5.1765	4.9845	5.6936
Mountains		4.3825	4.3244	3.9839	4.9553	4.6781
Chinese Garden		4.6375	5.7034	5.0167	5.0835	5.5842
Laurentian Library		4.9774	5.3432	5.2776	5.5504	5.7724
Arno River		4.8022	5.2405	5.0546	5.4673	5.4493
**Average**		**4.9102**	**5.2867**	**5.0640**	**5.3400**	**5.4606**

Red numbers indicate the best NIQMC value of each row.

## References

[1] Li S., Handa A., Zhang Y., Calway A. HDR Fusion: HDR SLAM using a low-cost auto-exposure RGB-D sensor. Proceedings of the 2016 Fourth International Conference on 3D Vision.

[2] Wei Z., Wen C.Y., Li Z.G. (2018). Local inverse tone mapping for scalable high dynamic range image coding. IEEE Trans. Circuits Syst. Video Technol..

[3] Ozcinar C., Lauga P., Valenzise G., Dufaux F. (2018). Spatio-temporal constrained tone mapping operator for HDR video compression. J. Vis. Commun. Image Represent..

[4] Mertens T., Kautz J., Reeth F.V. (2009). Exposure fusion: A simple and practical alternative to high dynamic range photography. Comput. Graph. Forum.

[5] Jung J., Ho Y. (2013). Low-bit depth-high-dynamic range image generation by blending differently exposed images. Iet Image Process..

[6] Ancuti C.O., Ancuti C., Vleeschouwer C., Bovik A.C. (2017). Single-scale fusion: An effective approach to merging images. IEEE Trans. Image Process..

[7] Kinoshita Y., Shiota S., Kiya H. Automatic exposure compensation for multi-exposure image fusion. Proceedings of the IEEE International Conference Image Processing.

[8] Liu S., Zhang Y. (2019). Detail-preserving underexposed image enhancement via optimal weighted multi-exposure fusion. IEEE Trans. Consum. Electron..

[9] Hayat N., Imran M. (2019). Ghost-free multi exposure image fusion technique using dense SIFT descriptor and guided filter. J. Vis. Commun. Image Represent..

[10] Kinoshita Y., Kiya H. (2019). Scene segmentation-based luminance adjustment for multi-exposure image fusion. IEEE Trans. Image Process..

[11] Ma K., Li H., Yong H., Wang Z., Meng D., Zhang L. (2017). Robust multi-exposure image fusion: A structural patch decomposition approach. IEEE Trans. Image Process..

[12] Ma K., Duanmu Z., Yeganeh H., Wang Z. (2018). Multi-exposure image fusion by optimizing a structural similarity index. IEEE Trans. Comput. Imaging.

[13] Li Z., Zheng J., Zhu Z., Yao W., Wu S. (2015). Weighted guided image filtering. IEEE Trans. Image Process..

[14] Li Z., Zheng J. (2018). Single image de-hazing using globally guided image filtering. IEEE Trans. Image Process..

[15] Liu Y., Zheng C., Zheng Q., Yuan H. (2018). Removing Monte Carlo noise using a Sobel operator and a guided image filter. Vis. Comput..

[16] Belyaev A., Fayolle P.A. (2018). Adaptive curvature-guided image filtering for structure + texture image decomposition. IEEE Trans. Image Process..

[17] Lu Z., Long B., Li K., Lu F. (2018). Effective guide image filtering for contrast enhancement. IEEE Signal Process. Lett..

[18] Du J., Li W., Xiao B. (2017). Anatomical-functional image fusion by information of interest in local laplacian filtering domain. IEEE Trans. Image Process..

[19] Zhang H., Patel V.M. Densely connected pyramid dehazing network. Proceedings of the 2018 IEEE/CVF Conference on Computer Vision and Pattern Recognition.

[20] Ancuti C., Ancuti C.O. Laplacian-guided image decolorization. Proceedings of the 2016 IEEE International Conference on Image Processing.

[21] Li Z., Wei Z., Wen C., Zheng J. (2017). Detail-enhanced multi-scale exposure fusion. IEEE Trans. Image Process..

[22] Kou F., Li Z., Wen C., Chen W. (2018). Edge-preserving smoothing pyramid based multi-scale exposure fusion. J. Vis. Commun. Image Represent..

[23] Kou F., Chen W., Wen C., Li Z. (2015). Gradient domain guided image filtering. IEEE Trans. Image Process..

[24] Wang Q., Chen W., Wu X., Li Z. (2019). Detail-enhanced multi-scale exposure fusion in YUV color space. IEEE Transactions on Circuits and Systems for Video TechnologyI.

[25] Singh V., Dev R., Dhar N.K., Agrawal P., Verma N.K. (2018). Adaptive type-2 fuzzy approach for filtering salt and pepper noise in grayscale images. IEEE Trans. Fuzzy Syst..

[26] Pham T.X., Siarry P., Oulhadj H. (2018). Integrating fuzzy entropy clustering with an improved PSO for MRI brain image segmentation. Appl. Soft Comput..

[27] Liu M., Zhou Z., Shang P., Xu D. (2019). Fuzzified image enhancement for deep learning in iris recognition. IEEE Trans. Fuzzy Syst..

[28] Celebi A.T., Duvar R., Urhan O. (2015). Fuzzy fusion based high dynamic range imaging using adaptive histogram separation. IEEE Trans. Consum. Electron..

[29] Rahman M.A., Liu S., Wong C.Y., Lin S.C.F., Liu S.C., Kwok N.M. (2017). Multi-focal image fusion using degree of focus and fuzzy logic. Digit. Signal Process..

[30] Chen Y., Hsia C., Lu C. (2019). Multiple exposure fusion based on sharpness-controllable fuzzy feedback. J. Intell. Fuzzy Syst..

[31] Lafferty J., McCallum A., Pereira F. Conditional Random Fields: Probabilistic Models for Segmenting and Labeling Sequence Data. Proceedings of the 18th International Conference on Machine Learning.

[32] Thakare B.S., Deshmkuh H.R. An Adaptive Approach for Image Denoising Using Pixel Classification and Gaussian Conditional Random Field Technique. Proceedings of the 2017 International Conference on Computing, Communication, Control and Automation.

[33] Li F.Y., Shafiee M.J., Chung A.G., Chwyl B., Kazemzadeh F., Wong A., Zelek J. High dynamic range map estimation via fully connected random fields with stochastic cliques. Proceedings of the 2015 IEEE International Conference on Image Processing.

[34] Fu K., Gu I.Y., Yang J. (2017). Saliency detection by fully learning a continuous conditional random field. IEEE Trans. Multimed..

[35] Sultani W., Mokhtari S., Yun H.B. (2018). Automatic pavement object detection using superpixel segmentation combined with conditional random field. IEEE Trans. Intell. Transp. Syst..

[36] Wang H.C., Lai Y.C., Cheng W.H., Cheng C.Y., Hua K.L. (2017). Background extraction based on joint Gaussian conditional random fields. IEEE Trans. Circuits Syst. Video Technol..

[37] Photomatix Database. https://www.hdrsoft.com/index.html.

[38] HDR Photography Gallery. https://www.easyhdr.com/examples/.

[39] Trivedi M., Jaiswal A., Bhateja V. A no-reference image quality index for contrast and Sharpness measurement. Proceedings of the 3rd IEEE International Advance Computing Conference (IACC).

[40] Ma K., Zeng K., Wang Z. (2015). Perceptual quality assessment for multi-exposure image fusion. IEEE Trans. Image Process..

[41] Zhang L., Zhang L., Bovik A. (2015). A feature-enriched completely blind image quality evaluator. IEEE Trans. Image Process..

[42] Gu K., Lin W., Zhai G., Yang X., Zhang W., Chen C. (2017). No-reference quality metric of contrast-distorted images based on information maximization. IEEE Trans. Cybern..

[43] Yang Y., Cao W., Wu S., Li Z. (2018). Multi-scale fusion of two large-exposure-ratio images. IEEE Signal Process. Lett..

